# Supplementary material for: The interactome of intact mitochondria by cross-linking mass spectrometry provides evidence for coexisting respiratory supercomplexes
Source: Mol Cell Proteomics. 2017 Dec 8;17(2):216–32. doi: 10.1074/mcp.RA117.000470 (PMC5795388; doi:10.1074/mcp.RA117.000470)
Supplement: Supplemental Data [file supp_RA117.000470_133922_0_supp_23978_fzffwf.zip › spectra_annotation/mito_spectra_annotation/51-1-5-1-7-1.pdf]

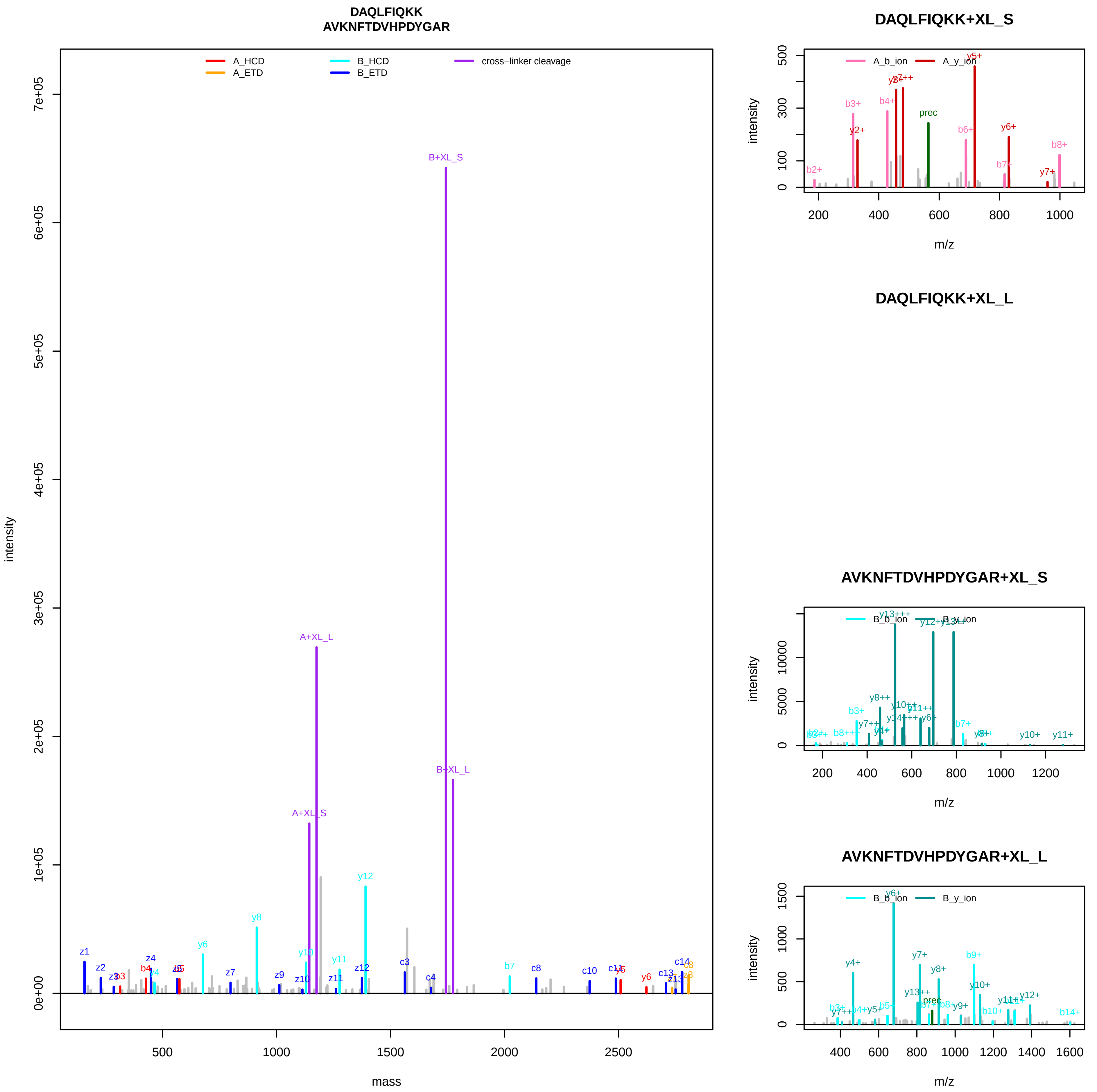
DAQLFIQKK
AVKNFTDVHPDYGAR
A_HCD
A_ETD
B_HCD
B_ETD
cross−linker cleavage
B+XL_S
A+XL_L
B+XL_L
A+XL_S
y12
y8
y6
y10
y11
z12
c3
c4
b7
c8
c10
c11
c13
c14
z13
y6
y5
z1
z2
z3
b3
b4
z4
y4
z5
b5
z7
z9
z10
z11
intensity
mass
0e+00
1e+05
2e+05
3e+05
4e+05
5e+05
6e+05
7e+05
500
1000
1500
2000
2500
DAQLFIQKK+XL_S
A_b_ion
A_y_ion
b2+
b3+
y2+
b4+
prec
b6+
y5+
y6+
b7+
y7+
b8+
m/z
DAQLFIQKK+XL_L
AVKNFTDVHPDYGAR+XL_S
B_b_ion
B_y_ion
y13+++
y8++
b3+
y7++
y4+
b7+
y10+
y11+
AVKNFTDVHPDYGAR+XL_L
y6+
y4+
y7+
b9+
y8+
y10+
y13++
prec
y9+
b10+
y12+
b14+
b3+
b5+
y5+
